# Comparative fluorimetric strategies for determination of landiolol hydrochloride using native fluorescence and green synthesized silver nanoparticles

**DOI:** 10.1038/s41598-026-63573-1

**Published:** 2026-07-28

**Authors:** Marwa Khaled, Hend Z. Yamani, Nermine V. Fares, Amira M. El-Kosasy

**Affiliations:** Pharmaceutical Analytical Chemistry Department, Faculty of Pharmacy, Ain Shams University, Cairo, 11566 Egypt

**Keywords:** Landiolol hydrochloride, Native fluorescence, Silver nanoparticles, Green synthesis, Greenness assessment, Blueness assessment, Biotechnology, Chemistry, Nanoscience and technology

## Abstract

This study introduces the first reported stepwise fluorimetric strategy for the sensitive and rapid determination of the ultra-short-acting β₁-blocker, landiolol hydrochloride. Initially, an environmentally benign method was developed by exploiting the drug’s native fluorescence in water (λ_ex_/λ_em_ = 217/298 nm). While this cost-effective approach successfully utilized water as a green solvent, it demonstrated limited LOD of 32.57 ng/mL. To enhance fluorescence performance, various organic solvents were investigated, with acetonitrile providing the highest signal at λ_ex_/λ_em_ = 222/300 nm and improving the LOD to 16.31 ng/mL. To enable ultra-trace clinical monitoring, a third “turn-on” method was developed using silver nanoparticles (AgNPs). By measuring fluorescence enhancement at λ_ex_/λ_em_ = 260/524.6 nm upon interaction with landiolol, this platform amplifies the signal, achieving an outstanding LOD of 3.10 ng/mL. AgNPs were prepared using a green synthesis approach employing *Aloe vera* extract as a natural reducing and stabilizing agent in an aqueous medium avoiding using hazardous chemicals. Water was used throughout both nanoparticle preparation and the AgNP-enhanced determination, reinforcing the eco-friendly profile of the method. Unlike native fluorescence, the AgNP-assisted method enhances sensitivity through nanoparticle-mediated surface passivation, with a possible auxiliary contribution from resonance energy transfer (RET)-like processes. Selectivity is improved through preferential adsorption of landiolol on the AgNP surface and a large Stokes shift that minimizes interference from UV-absorbing matrix components. The resulting enhanced sensitivity and reduced matrix interference make this method a powerful tool for ultra-trace, selective, and sustainable determination of landiolol in pharmaceutical formulations and human plasma, demonstrating its suitability for routine quality control and bioanalytical applications.

**Supplementary Information:**

The online version contains supplementary material available at 10.1038/s41598-026-63573-1.

## Introduction

Landiolol is an ultra-short-acting, highly selective β1-adrenergic receptor blocker. Japan approved it for the intraoperative tachyarrhythmia treatment in 2002. It is now widely used to treat perioperative tachycardia, especially atrial fibrillation, and tachyarrhythmias in both cardiac and noncardiac surgeries^1^. In November 2024, it received FDA approval for short-term decrease of ventricular rate in individuals with supraventricular tachycardia, which includes atrial fibrillation and atrial flutter, in critical care settings^2^.

High dose of landiolol can cause significant cardiovascular and central nervous system toxicity^3^. The clinical effect and patient safety are managed in real-time through bedside monitoring and dose titration, making the measurement of specific landiolol blood levels redundant in the standard treatment of supraventricular tachycardia^4^. Its therapeutic efficacy is highly dose-dependent, making accurate monitoring of its concentration in pharmaceutical formulations and biological matrices crucial.

Various analytical techniques have been reported for the determination of landiolol, most notably chromatographic methods including high-performance liquid chromatography (HPLC) with UV^5^, fluorescence^6,7^ and mass spectrometric (LC-MS/MS) detection^8^. These techniques offer high accuracy and selectivity, but they are time-consuming, require costly, complex instruments, demand extensive sample preparation, and rely on expert operators. Furthermore, the large quantities of organic solvents they use create environmental and safety issues^9^. In contrast, fluorimetric methods present a compelling substitute. They combine simplicity, speed, and cost-effectiveness, allowing for the detection of extremely low analyte concentrations (nanomolar range) with minimal preparation^10^. Their eco-friendly nature and suitability for high-throughput workflow make them ideal for routine quality control and biological drug monitoring, especially in resource-constrained settings.

Methods relying on the native fluorescence of drugs offer several analytical advantages. They do not require derivatization, resulting in a simple, rapid, low-cost workflow. Native fluorescence also provides good selectivity, since only intrinsically fluorescent compounds generate a measurable signal, thereby minimizing interference from non-fluorescent matrix components^11^.

Silver nanoparticles (AgNPs) possess unique optical, and electronic properties, including strong localized surface plasmon resonance (LSPR), high surface-to-volume ratio, and tunable fluorescence enhancement, which make them excellent candidates for sensitive and selective fluorimetric detection of drugs^12,13^. AgNPs can be synthesized via chemical, physical, or biological routes. While conventional chemical and physical methods are widely used, they are often limited by high energy consumption, the use of hazardous solvents, and the requirement for rigorous purification steps. In contrast, biological synthesis using plant extracts—often termed ‘green synthesis’—has emerged as a superior alternative. This approach is not only cost-effective and energy-efficient but also eliminates the need for toxic chemicals, as naturally occurring phytochemicals act as reducing, stabilizing and capping agents^14^. Consequently, this eco-friendly method yields biocompatible nanoparticles suitable for analytical applications without the environmental burden of traditional techniques^15^.

The sensing principle is based on the turn-on fluorescence enhancement of *Aloe vera*-capped AgNPs upon interaction with landiolol. These nanoparticles are naturally coated with an oxygen-rich phytochemical layer, which provides a surface with high electron density^16,17^. However, intrinsic surface defects on these nanoparticles act as non-radiative trap states, limiting their fluorescence efficiency^18^. Upon addition of landiolol, its cationic amine groups interact electrostatically and through hydrogen bonding with surface carboxylate and hydroxyl moieties of the capped AgNPs^19,20^. This interaction passivates surface defects, suppresses non-radiative relaxation pathways, and promotes radiative recombination, leading to enhanced fluorescence intensity^21^. Furthermore, the partial spectral overlap between landiolol emission and the AgNP surface plasmon resonance (SPR) band suggests a possible secondary contribution to RET-like process, in which landiolol acts as an energy donor transferring energy to the nanoparticles to contribute to the overall fluorescence enhancement^22,23^. The fluorescence enhancement is primarily governed by surface passivation of AgNP defect states by landiolol, while any energy-transfer process may provide an auxiliary contribution to the overall signal enhancement.

**Fig. 1:**
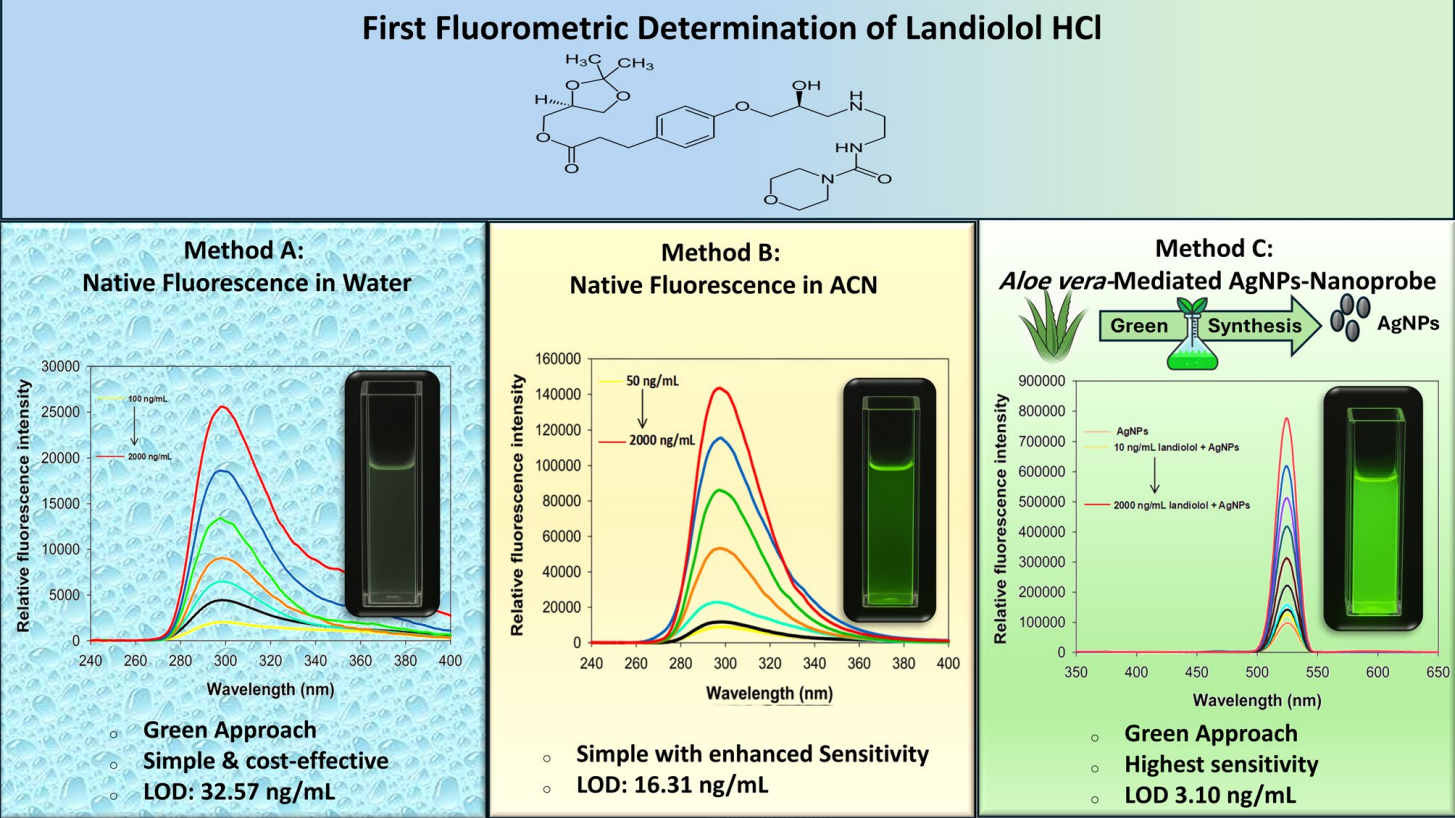
Stepwise fluorimetric strategy for enhancing the analytical performance in the determination of landiolol hydrochloride.

To the best of our knowledge, no fluorimetric methods have been reported for the determination of landiolol hydrochloride. This study aims to develop a novel, stepwise fluorimetric strategy to enhance analytical performance for the determination of landiolol hydrochloride in pharmaceutical dosage forms and plasma. The study establishes methods based on the drug’s native fluorescence as well as a highly sensitive ‘Turn-On’ nano probe strategy utilizing *Aloe vera*-mediated AgNPs as presented in Fig. 1. By exploiting the interaction between the drug and the bio-functionalized nanoparticles, the method primarily relies on surface passivation of AgNP defect states, with a possible secondary contribution from energy-transfer–like processes. This approach achieves an optimal balance between high sensitivity, operational simplicity, and environmental sustainability.

## Experimental

### Chemicals and reagents

Analytical-grade reagents and chemicals were sourced for all experimental procedures. Landiolol hydrochloride standard was procured from Baoji Guokang Bio-Technology (China). Rapiblyk^®^ injection was obtained from AOP Orphan Pharmaceuticals GmbH (Austria). Boric acid, creatinine, glacial acetic acid, leucine, 85% ortho-phosphoric acid, and silver nitrate were obtained from Sigma-Aldrich (Germany). Acetonitrile, ammonium acetate, and absolute ethanol, methanol, N, N-dimethyl-formamide (DMF) were purchased from Merck (Germany). DL-alanine was obtained from S.D. Fine-Chem Ltd. (India), and L-arginine from WINLAB (Pakistan). Hydrochloric acid, sodium hydroxide, glucose, urea, sodium chloride, sodium bicarbonate, sodium carbonate, potassium dihydrogen phosphate, disodium hydrogen phosphate were obtained from Prolabo (USA). *Aloe vera* leaves were purchased from Shatla Sky store. Plasma samples were purchased from VACSERA (Egypt). Deionized water was employed for all experiments.

### Instrumentation

Surface morphology was investigated using a Quanta FEG 250 scanning electron microscope (SEM) (FEI, USA), while elemental composition was verified by energy-dispersive X-ray analysis (EDAX). The particle size distribution, morphology, and crystallinity were further characterized using a JEM-2100 high-resolution transmission electron microscope (HR-TEM) (JEOL, Japan) operated at an accelerating voltage of 200 kV. Fourier transform infrared (FTIR) spectra were collected to determine the functional groups present, employing a Vertex 80 V FTIR spectrometer (Bruker, Germany). UV–Visible absorption measurements were conducted with a 1601 PC double-beam UV–Vis spectrophotometer (Shimadzu, Japan). Fluorescence analyses were performed using a Shimadzu RF-6000 spectrofluoriphotometer (Japan) equipped with an R928 photomultiplier tube and 3D scanning capability. Measurements were obtained in 1 cm quartz cells and controlled through LabSolutions RF software. Both excitation and emission slit widths were adjusted to 10 nm. Spectra were recorded at a scanning rate of 600 nm min⁻¹ with a data acquisition interval of 0.2 nm.

### Procedures

#### Green AgNPs synthesis procedure

AgNPs were synthesized via a modified green route adapted from Anju et al.^16^ and Logaranjan et al.^17^. While the reference method relied on the inner gel and required the addition of sodium borohydride (NaBH₄) to catalyze the reduction, our protocol was optimized to utilize a hot water extraction of the whole leaf. This modification was designed to extract the potent phenolic reducing agents (e.g., anthraquinones) present in the leaf rind, thereby eliminating the need for external chemical catalysts. Consequently, a purely thermal reduction strategy was employed, using the extract as the sole reducing and stabilizing agent to ensure a fully eco-friendly synthesis.

##### Preparation of *Aloe vera* extract

To prepare the extract, 30 g of fresh *Aloe vera* leaves were thoroughly washed with deionized water and finely chopped to maximize surface area. The plant material was then subjected to hot aqueous extraction by boiling in 50 mL of deionized water for 20 min. This thermal extraction step ensures the release of active phytochemicals required for the reduction process. After cooling, the resulting leaf broth was filtered to remove solid residues. The final liquid extract was stored at 4 °C in a refrigerator until use.

##### Synthesis of AgNPs

A 0.2 M silver nitrate (AgNO₃) solution was prepared by dissolving the salt in 30 mL of deionized water. This solution was mixed with 30 mL of the prepared *Aloe vera* extract (1:1 v/v) under vigorous stirring at room temperature for 30 min to ensure a homogenous mixture. To drive the reduction of silver ions, the mixture was heated in an open glass vessel on a hot plate under atmospheric pressure for 25 min. The hot plate was set to 150 °C, while the aqueous reaction mixture was maintained near its boiling temperature. A visible color transition from yellow to dark brown confirmed the successful formation of AgNPs, stabilized by the *Aloe vera* matrix without external chemical catalysts. The prepared Ag NPs were stored at 4 °C in the dark.

#### Preparation of standard solutions

By dissolving landiolol hydrochloride in deionized water, a standard stock solution of the drug (100 µg/mL) was created. By diluting the stock solution, a working solution was produced at a concentration of 10 µg/mL.

#### General spectrofluorimetric procedures and construction of calibration plots

##### Method A (Native fluorescence in water)

Aliquots of landiolol hydrochloride working standard solution (10 µg/mL) were dispensed into a series of 5 mL volumetric flasks. The final volume was completed with deionized water to achieve concentrations between 100 and 2000 ng/mL. Fluorescence was recorded at excitation and emission wavelengths of 217 nm and 298 nm, respectively. A blank experiment was carried out alongside the main study. A regression equation was then derived from a calibration curve correlating corrected fluorescence intensities with final drug concentrations.

##### Method B (Native fluorescence in acetonitrile)

Accurately measured volumes of landiolol hydrochloride working standard solution (10 µg/mL) were transferred into a series of 5 mL volumetric flasks. The final volume was completed with acetonitrile to obtain concentrations in the range of 50–2000 ng/mL. Fluorescence was recorded at excitation and emission wavelengths of 222 nm and 300 nm, respectively. A blank experiment was carried out alongside the main study. A regression equation was then derived from a calibration curve correlating corrected fluorescence intensities with final drug concentrations.

##### Method C (AgNP-enhanced fluorescence assay in water)

To prepare the calibration standards, precise aliquots of landiolol hydrochloride working solution (10 µg/mL) were combined with 5 µL of silver nanoparticles (AgNPs) in a series of 5 mL volumetric flasks. The mixtures were diluted to volume with deionized water to yield a concentration gradient ranging from 10 to 2000 ng/mL. Fluorescence measurements were performed immediately at excitation and emission wavelengths of 260 and 524.6 nm, respectively. A reagent blank was analyzed under identical conditions. The analytical calibration curve was constructed by plotting the relative fluorescence enhancement, defined by the ratio I/I_0_, against the final drug concentration. (where *I* is the fluorescence intensity of AgNPs after landiolol addition, and *I*_0_ is the fluorescence intensity of AgNPs alone) against the final drug concentrations, and the regression equation was computed.

#### Determination of landiolol hydrochloride in the pharmaceutical dosage form procedure

Rapiblyk^®^ injection was reconstituted with 50 mL of 5% dextrose injection, USP. An accurately measured aliquot of the reconstituted injection was transferred into a 100 mL-volumetric flask, and the volume was completed with deionized water to prepare an intermediate solution of 100 µg/mL. Aliquots of the intermediate solution were quantitatively transferred into 5 mL volumetric flasks. The final volume was completed with deionized water for (method A), with acetonitrile (method B), and for (method C), 5 µL of AgNPs was first added, then the final volume was completed with deionized water to obtain concentrations of 700, 400, and 200 ng/mL. The samples were then analyzed as described under the respective procedure for the calibration curve. The standard addition technique was performed by spiking the pre-analyzed dosage form (200 ng/mL) with landiolol hydrochloride at three distinct levels (50, 100 and 150%). This approach was utilized to assess the influence of the sample matrix on the analyte’s recovery.

#### Determination of landiolol hydrochloride in human plasma procedure

Human plasma aliquots (950 µL) were transferred into centrifuge tubes and spiked with 50 µL of the appropriate landiolol hydrochloride stock solution at different levels covering the C_max_ and lower therapeutic levels (1770, 1000, 700, 500, and 200 ng/mL). The tubes were vortexed for 30 s. Protein precipitation was subsequently induced by the addition of 4 mL of cold acetonitrile. The samples were then centrifuged at 6000 rpm for 10 min, and the resulting supernatant was collected and evaporated at a controlled temperature of 40 °C. The residue was reconstituted with 1.0 mL of deionized water (for methods A and C) or 1.0 mL of acetonitrile (for method B), and quantitatively transferred into a series of 5-mL volumetric flasks. For method A, the final volume was completed with deionized water; for method B, with acetonitrile; and for method C, 5 µL of AgNPs was first added before completing the final volume with deionized water. The samples were then analyzed using procedure described in the calibration curve section.

## Results and discussion

Fluorimetric methods represent a powerful and compelling analytical alternative because they are highly sensitive, rapid, simple, and cost-effective, enabling the quantification of extremely low analyte concentrations, down to the nanomolar range, with minimal sample preparation. A sequential fluorimetric approach was successfully created for the sensitive and rapid detection of landiolol hydrochloride. This strategy demonstrated continuously improving analytical performance.

A slit width of 10 nm was selected as it provided an optimal balance between fluorescence signal intensity and spectral resolution, resulting in an improved signal-to-noise ratio and enhanced sensitivity for quantitative measurements.

Initially, a straightforward, low-cost, and environmentally friendly approach was established by measuring the drug’s native fluorescence in water at λ_ex_/λ_em_ = 217/298 nm (**Fig. ****S1****a**). Water was chosen as the main solvent due to its availability, safety, and green characteristics. While this method successfully characterized the intrinsic fluorescence of the drug, its sensitivity was limited, with a LOD of 32.57 ng/mL. To enhance the fluorescence signal, several organic solvents were tested, and acetonitrile was found to produce the highest fluorescence intensity at λ_ex_/λ_em_ = 222/300 nm (**Fig. ****S1****b**). This allowed the development of a second method in acetonitrile, reducing the LOD to 16.31 ng/mL.

To achieve ultra-trace sensitivity, a third strategy was implemented utilizing *Aloe vera*-mediated AgNPs as active fluorogenic probes. This method capitalized on the significant enhancement of the intrinsic nanoparticle emission at λ_ex_/λ_em_ = 260/524.6 nm (**Fig. ****S1****c**) triggered by the interaction with landiolol. The AgNPs were synthesized using *Aloe vera* extract in an aqueous medium, offering green and sustainable preparation. Water was used throughout both the nanoparticle synthesis and the analytical determination, preserving the overall eco-friendly nature of the methodology while achieving the highest sensitivity (LOD of 3.10 ng/mL).

### Synthesis of AgNPs

Green synthesis of AgNPs using *Aloe vera* is an environmentally friendly method in which the plant’s bioactive constituents serve simultaneously as reducing and capping agents. This approach offers a cost-effective, non-toxic alternative to traditional chemical methods, as it avoids harmful solvents and byproducts. The process typically involves reacting to silver nitrate with a biological extract, causing a change from pale yellow to brown as the AgNPs form. This characteristic coloration is the specific optical signature of the formation of metallic silver (Ag⁰), arising from the excitation of Surface Plasmon Resonance (SPR) in the nanoparticles.

Mechanistically, the *Aloe vera* extract contains phytochemicals (specifically polyphenols and anthraquinones from the leaf rind and polysaccharides from the gel) that serve as natural reducing agents. These compounds effectively reduce Ag⁺ ions into metallic Ag⁰ nanoparticles without added chemicals, a reaction efficiently driven by the thermal activation employed in our method. Furthermore, the large macromolecular chains like polysaccharides and proteins present in the whole-leaf extract physically surround the nanoparticle acting as capping and stabilizing agents^14,24^. The adsorbed phytochemical layer provides steric stabilization, helping to minimize particle aggregation and surface oxidation and thereby preserve nanoparticle stability during storage. To verify the stability of the prepared AgNPs, the nanoparticles were stored at 4 °C in the dark and their fluorescence-enhancing performance was monitored over a period of 28 days. As shown in **Figure S2**, the relative fluorescence enhancement response (I/I₀) of the landiolol–AgNPs system remained highly stable throughout the study period, demonstrating that the biogenic AgNPs retained their analytical performance under the investigated storage conditions.

To confirm the reproducibility of the synthesis process, the relative fluorescence enhancement ratio (I/I₀) was evaluated using two independently synthesized AgNP batches. The excellent agreement between batches, reflected by an inter-batch RSD of 1.703%, demonstrates the reproducibility of both the nanoparticle synthesis and the analytical response.

### Characterization of AgNPs

#### Scanning electron microscopy (SEM) and energy dispersive x-ray analysis (EDAX)

**Fig. 2:**
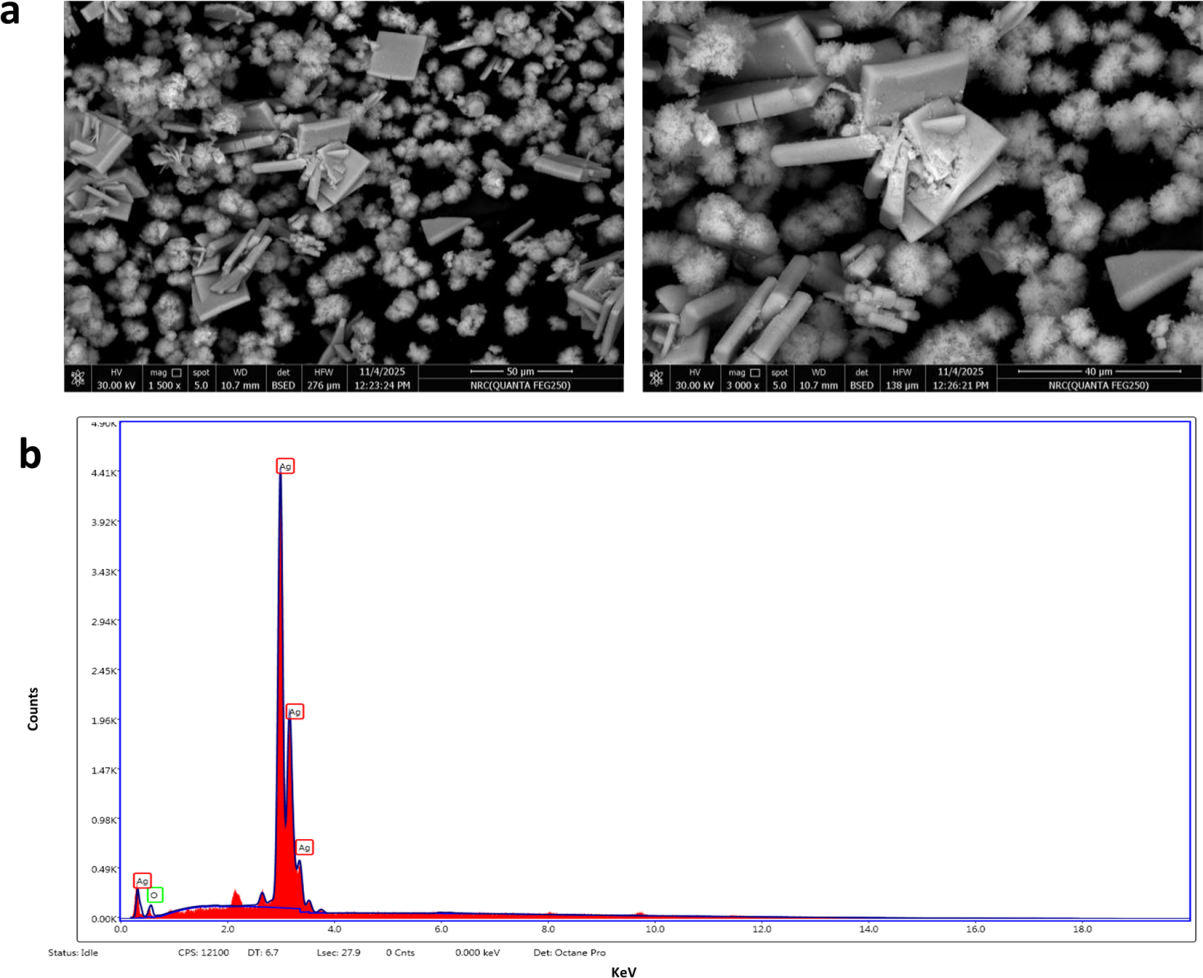
(a) SEM images and (b) EDAX spectrum of the synthesized AgNPs.

Scanning electron microscopy (SEM) was employed to evaluate the surface morphology of the synthesized silver nanoparticles (AgNPs) as shown in Fig. 2a. The images reveal a complex morphology typical of biogenic synthesis, where the nanoparticles are embedded within a visible biological scaffold with polymorphic structures. AgNPs exhibit spherical aggregates that appear as dense clumps of much smaller primary particles. This characteristic ‘cauliflower-like’ aggregation is not a sign of uncontrolled crystal growth, but rather the result of the binding effect of the organic matrix-derived from the whole leaf extract-upon drying. This organic encapsulation is a critical feature, as it stabilizes the high surface energy of the primary nanoparticles. Additionally, distinct triangular and tetragonal structures are also observed within the spherical population. This structural diversity is typical of plant-mediated synthesis, where different phytochemicals in the whole leaf extract modulate crystal growth rates along different facets.

EDAX analysis (Fig. 2b) revealed a dominant peak for Ag at ~ 3 keV along with additional metallic silver nanocrystallites (Ag L-series) peaks, confirming the successful reduction of silver ions to metallic silver. Notably, a minor oxygen peak was also detected, which may be attributed to surface-bound organic capping agents derived from the *Aloe vera* phytochemicals further verifying the core-shell structure implied by the morphological analysis.

#### Transmission electron microscopy (TEM)

**Fig. 3:**
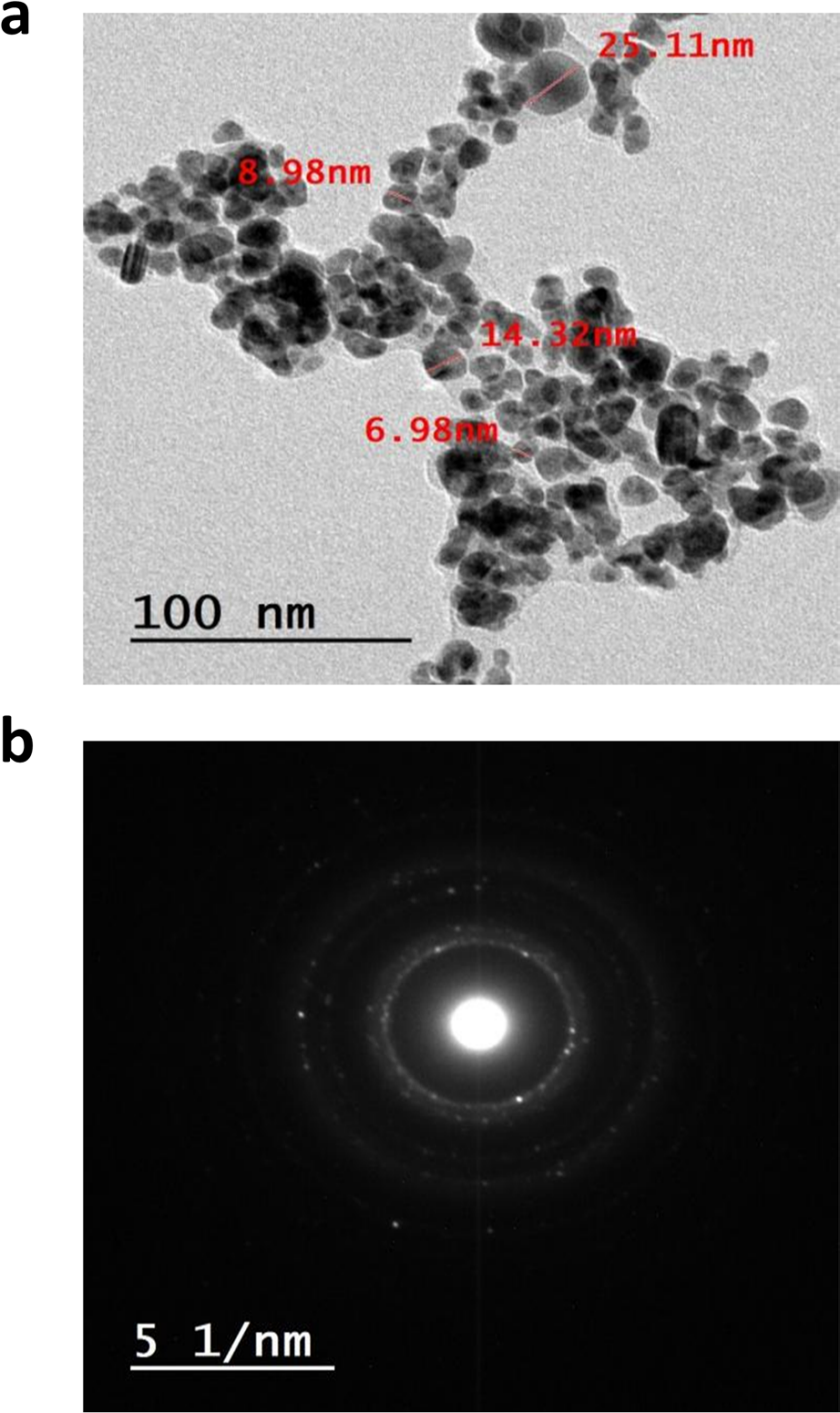
(a) HRTEM images and (b) SAED pattern of the synthesized AgNPs.

TEM is the gold standard for directly visualizing the morphology and size of primary nanoparticles overcoming the aggregation artifacts observed in SEM. As presented in Fig. 3a, the primary particles appear predominantly spherical to near-spherical in shape, with particle sizes well within the nanoscale range (< 100 nm) and a significant population of smaller clusters (< 25 nm). A few slightly elongated structures are also observed.

This predominant spherical morphology provides evidence for the stabilizing role of the polysaccharides abundantly present in the whole leaf extract, which act as steric capping agents to restrict anisotropic growth. Furthermore, the prevalence of small particle sizes (< 25 nm) validates the effectiveness of the thermal activation strategy; the rapid reduction kinetics at elevated temperatures favor burst nucleation, generating a high density of small nuclei rather than large crystals. This high surface-to-volume ratio is advantageous for our probe’s performance, providing a maximal number of active sites for the subsequent interaction with landiolol.

The selected area electron diffraction (SAED) (Fig. 3b) shows distinct concentric diffraction rings corresponding to the (111), (200), (220), (311), and (331) planes of face-centered cubic structure (FCC) lattice of metallic silver (ICDD card No. 04–0783). The clarity of these rings, rather than a single spot or a broad halo, confirms that, despite the rapid green synthesis, the resulting AgNPs possess a highly crystalline metallic nature^25^.

#### FT-IR analysis

Fourier-transform infrared spectroscopy (FTIR) was employed to determine the functional groups of the bioactive capping layer from the *Aloe vera* extract that are responsible for reducing Ag⁺ to Ag⁰ and stabilizing (capping) the resulting AgNPs. This characterization is crucial for understanding the sensing mechanism, as these surface groups serve as the docking sites for the analyte.

**Fig. 4:**
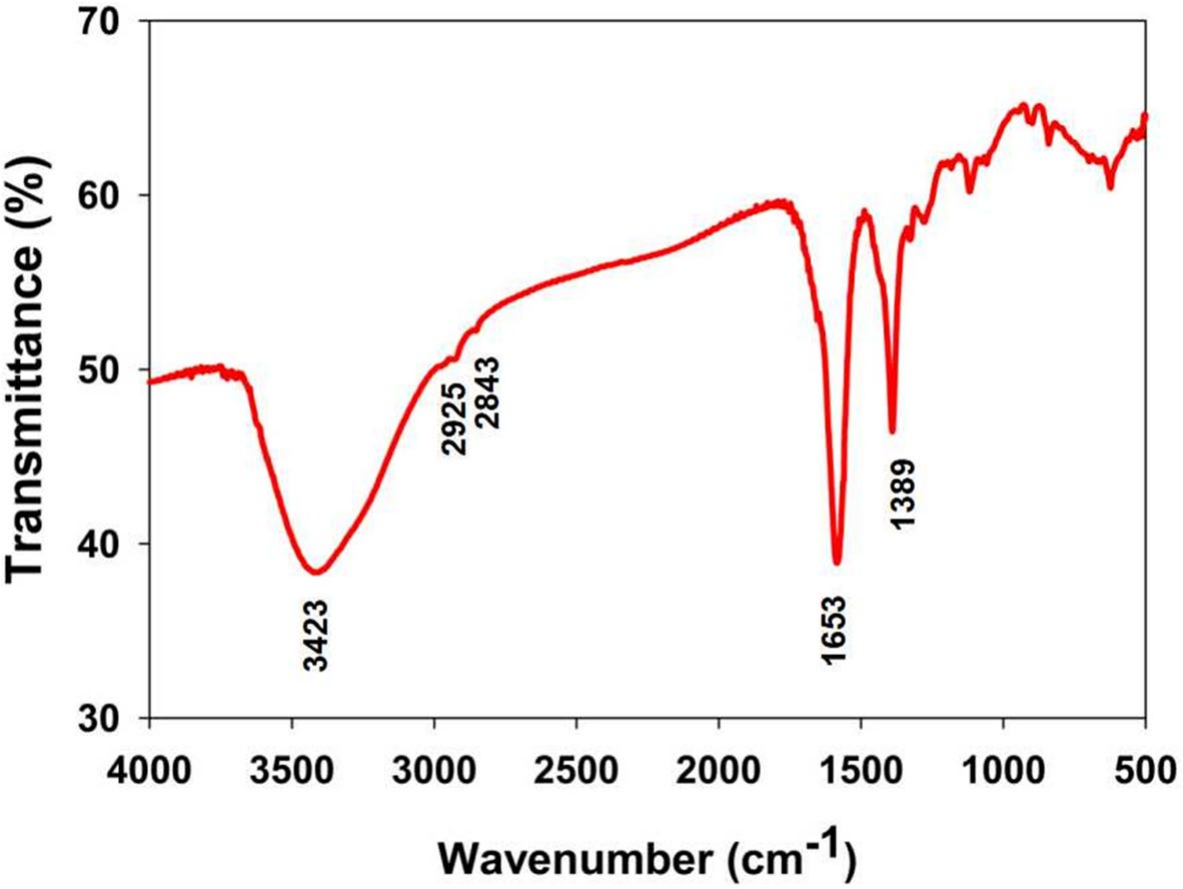
FT-IR spectrum of AgNPs.

In the FTIR spectrum presented in Fig. 4, the broad absorption band observed at 3423 cm^− 1^ is attributed to the stretching vibrations of O-H bonds that confirm the presence of extensive intermolecular hydrogen bonding on AgNPs surface. The peaks at 2925 and 2843 cm^− 1^ correspond to C-H stretching vibrations of the aliphatic backbone. A prominent sharp peak at 1653 cm^− 1^ may due to the asymmetrical –COO⁻ stretching of carboxylate groups in *Aloe vera.* This peak is chemically significant as it serves as a fingerprint for the presence of both the acetylated polysaccharides (acemannan) from the gel and the anthraquinones (such as aloin) extracted from the leaf rind^16,17^. A sharp peak at 1389 cm^− 1^ corresponds to C-H bending vibrations. Multiple strong peaks in the 1200–900 cm^− 1^ region are characteristic of the C-O stretching vibrations (C-O-C and C-O-H) of the polysaccharide backbone^16^. These results confirm that the green-synthesized AgNPs are capped by a dense, oxygen-rich organic layer derived from *Aloe vera* phytochemicals. The abundance of electron-rich functional groups (hydroxyls, carbonyls, and carboxylates) creates a high electron density surface that facilitates the adsorption of the amine-containing landiolol through intermolecular hydrogen bonding and electrostatic interactions^26^, thereby enabling close-proximity interactions necessary for surface-mediated passivation and RET-like process.

### Optimization of experimental conditions for the AgNP-enhanced fluorescence method

#### Effect of AgNPs volume

**Fig. 5:**
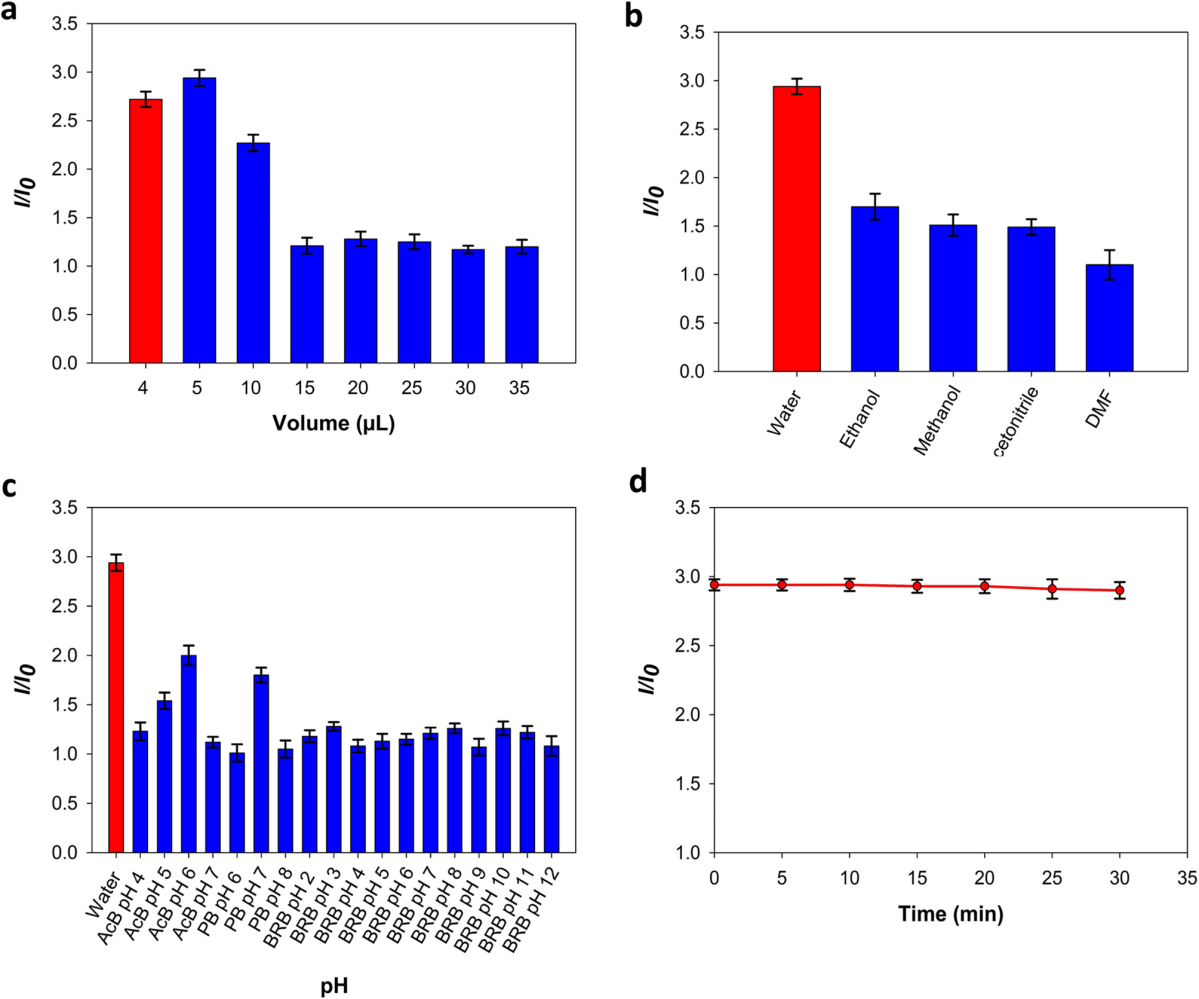
Optimization of different experimental conditions affecting fluorescence enhancement by AgNPs: (a) volume of AgNPs, (b) solvents, (c) pH, and (d) incubation time.

The volume of the colloidal AgNPs added to the reaction mixture plays a critical role in maximizing the sensitivity of the probe. Different volumes ranging from 4 µL to 35 µL of AgNPs were investigated. As shown in (Fig. 5a**)**, the fluorescence enhancement (*I/I*₀) increased sharply to reach a maximum at 5 µL of AgNPs. This initial enhancement is ascribed to the augmented accessibility of surface binding sites for landiolol. However, increasing the volume beyond 5 µL resulted in a marked decrease in fluorescence enhancement. This quenching phenomenon may be primarily attributed to the Inner Filter Effect (IFE). At higher concentrations, the dense cloud of nanoparticles absorbs a significant portion of both the excitation light (260 nm) and the emitted light (524.6 nm) before it reaches the detector, effectively masking the signal^27^. Additionally, excessive particle density and probable aggregation lead to light scattering, which interferes with the fluorescence measurement^28^.

#### Effect of different solvents

The physicochemical environment of the reaction medium significantly influences the interaction between the drug and the nanoparticles. The effect of different solvents was investigated using deionized water, ethanol, methanol, acetonitrile, and DMF. As demonstrated in Fig. 5b, the maximum fluorescence enhancement was observed in water, whereas the signal intensity notably decreased in the order of water > ethanol > methanol > acetonitrile > DMF.

The superior sensitivity observed in aqueous media is attributed to two factors. First, the stability of the capping layer: the *Aloe vera-*derived phytochemicals (polysaccharides and anthraquinones) stabilizing the AgNPs are inherently hydrophilic. Water maintains this capping layer in a hydrated conformation, preserving an electrical double layer and ensuring excellent colloidal stability. In contrast, adding organic solvents lowers the dielectric constant of the medium and dehydrates the capping shell. This induces the collapse of the protective electrical double layer, leading to particle destabilization and aggregation. Aggregated particles suffer from reduced surface area and increased light scattering, which diminishes the fluorescence signal^29^. Second, the binding efficiency: The proposed sensitization mechanism relies on the electrostatic attraction and hydrogen bonding between the landiolol amine group and the carboxyl/hydroxyl groups of the capping layer. This promotes the adsorption of landiolol onto the nanoparticle surface, which is essential for both effective surface passivation and establishing the close donor-acceptor proximity necessary for RET. Conversely, organic solvents diminish dielectric screening^30^ and disrupt non-covalent interactions. Consequently, water was selected as the optimal solvent, reinforcing the green and eco-friendly nature of the developed method.

#### Effect of pH

The effect of pH on fluorescence enhancement was investigated using different buffer systems, including ammonium acetate buffer (AcB) (pH 4–7), phosphate buffer (PB) (pH 6–8), and Britton-Robinson (BR) buffer (pH 2–12). As shown in Fig. 5c, none of the tested buffers were superior to deionized water in enhancing fluorescence. In fact, the introduction of buffer solutions resulted in a marked decrease in the analytical signal compared to the unadjusted aqueous medium. This signal reduction may be attributed to the high ionic strength of buffers, which compresses the electrical double layer surrounding the nanoparticles and diminishes electrostatic repulsion, as described by El Badawy et al.^31^. Consequently, this reduction in repulsive forces facilitates particle aggregation^32^, making the unadjusted aqueous medium the optimal choice for maintaining colloidal stability and maximizing the sensitized fluorescence signal.

The pH of the unadjusted mixture containing AgNPs, landiolol, and water was measured using a calibrated pH meter and found to be 6.28 ± 0.015. At this pH environment, the surface carboxylate functionalities of the *Aloe vera* capping layer are expected to be predominantly deprotonated, while the secondary amine groups of landiolol are fully protonated into a highly cationic form. This optimizes the electrostatic attraction and hydrogen bonding required for close-proximity surface passivation. Additionally, the biogenic capping layer derived from *Aloe vera* extract provided steric stabilization to the AgNPs, reducing their susceptibility to aggregation under these unbuffered conditions. The AgNP dispersion was visually monitored during the analytical time window, and no signs of aggregation or turbidity changes were observed. Ultimately, any risk of time-dependent pH drift or colloidal instability was minimized by preparing the samples freshly, maintaining constant reagent-to-sample ratios, and performing the fluorescence measurements immediately after preparation.

For plasma analysis, matrix-induced pH variations and nanoparticle aggregation were minimized by the sample pre-treatment procedure, which removed endogenous proteins and major buffering components. Subsequent reconstitution and dilution in deionized water ensured that the AgNP–landiolol interaction occurred in a controlled, low-ionic-strength medium. These conditions are expected to maintain colloidal stability without the need for an external buffer system.

#### Effect of incubation time

The incubation time was investigated to reach the highest and most stable fluorescence enhancement over a range of 0–30 min at 5 min intervals. The reaction was found to be rapid, achieving maximum fluorescence intensity immediately upon mixing. The signal remained stable for up to 20 min, providing a sufficient time window for convenient analysis. However, after 25 and 30 min, a minimal decrease in fluorescence intensity was observed, as shown in (Fig. 5d). Therefore, measurements were carried out immediately after mixing.

### Proposed mechanism of fluorescence enhancement

The excitation and emission characteristics of silver nanoparticles are highly dependent on particle aggregation state, and surface functionalization, including the nature of capping agents, which collectively govern their optical response and spectral behavior^33,34^.

The observed fluorescence enhancement can be rationalized through a stepwise surface interaction process. First, the AgNPs are capped by an oxygen-rich layer of *Aloe vera* phytochemicals, creating a surface with high electron density as indicated by the FTIR analysis (Sect. 3.2.3)^16,17^,. Upon the addition of landiolol, we propose that its cationic amine groups interact with these surface hydroxyl and carboxylate moieties through electrostatic and hydrogen-bonding forces further supported by the solvent optimization study (Sect. 3.3.2)^19,20^. Mechanistically, the AgNPs possess intrinsic surface defects^18^ that act as surface trap states, which often act as non-radiative recombination centers facilitating non-radiative relaxation. The adsorption of landiolol likely passivates these surface defects, reducing non-radiative relaxation pathways and allowing more electrons to recombine radiatively. This stabilization of the emissive state results in a marked increase in fluorescence intensity without altering the emission maximum^21^. This mechanism is further supported by the mirror-image relationship between the excitation and emission spectra which, along with concentration-dependent fluorescence enhancement and lack of spectral shifts indicate that the emissive state remains structurally stable upon interaction.

Additionally, upon excitation at λₑₓ = 260 nm, landiolol may act as an energy donor in a RET-like process to the proximal silver species. This interpretation is supported by the partial spectral overlap between the landiolol emission and the broad surface plasmon resonance (SPR) absorption band of the AgNPs (**Fig. S3**). Consistent with this mechanism, partial quenching of the intrinsic landiolol emission accompanied by enhanced AgNP fluorescence was observed^22,23^, indicating a possible synergistic effect, where surface passivation stabilizes the emissive states and energy transfer may further contribute to the fluorescence enhancement.

Overall, surface passivation of AgNP surface defect states is considered the primary mechanism responsible for the fluorescence enhancement, while the RET-like process provides a secondary, auxiliary contribution under conditions of spectral overlap.

### Method validation

The proposed methodologies were validated in accordance with ICH recommendations^35^. The parameters of validation are summarized in (Table 1).

**Table 1:** Validation parameters of the proposed methods for the determination of landiolol hydrochloride.

Parameter	Method A	Method B	Method C
**Excitation wavelength (nm)**	217	222	260
**Emission wavelength (nm)**	298	300	524.6
**Linearity range (ng/mL)**	100–2000	50–2000	10–2000
**Slope**	12.125	69.848	0.0033
**Intercept**	604.86	5126.5	1.2674
**Correlation coefficient (** ***r*** **)**	0.9998	0.9999	0.9999
**LOD (ng/mL)**	32.57	16.31	3.10
**LOQ (ng/mL)**	98.71	49.41	9.40
**Accuracy (mean ± SD)** ^*^	99.82 ± 0.196	99.27 ± 0.684	100.61 ± 0.215
**Intraday precision (%RSD)** ^*^	0.196%	0.684%	0.213%
**Inter-day precision (%RSD)** ^*^	0.493%	0.823%	0.379%

^*^Average of 3 replicates.

#### Linearity and sensitivity

**Fig. 6:**
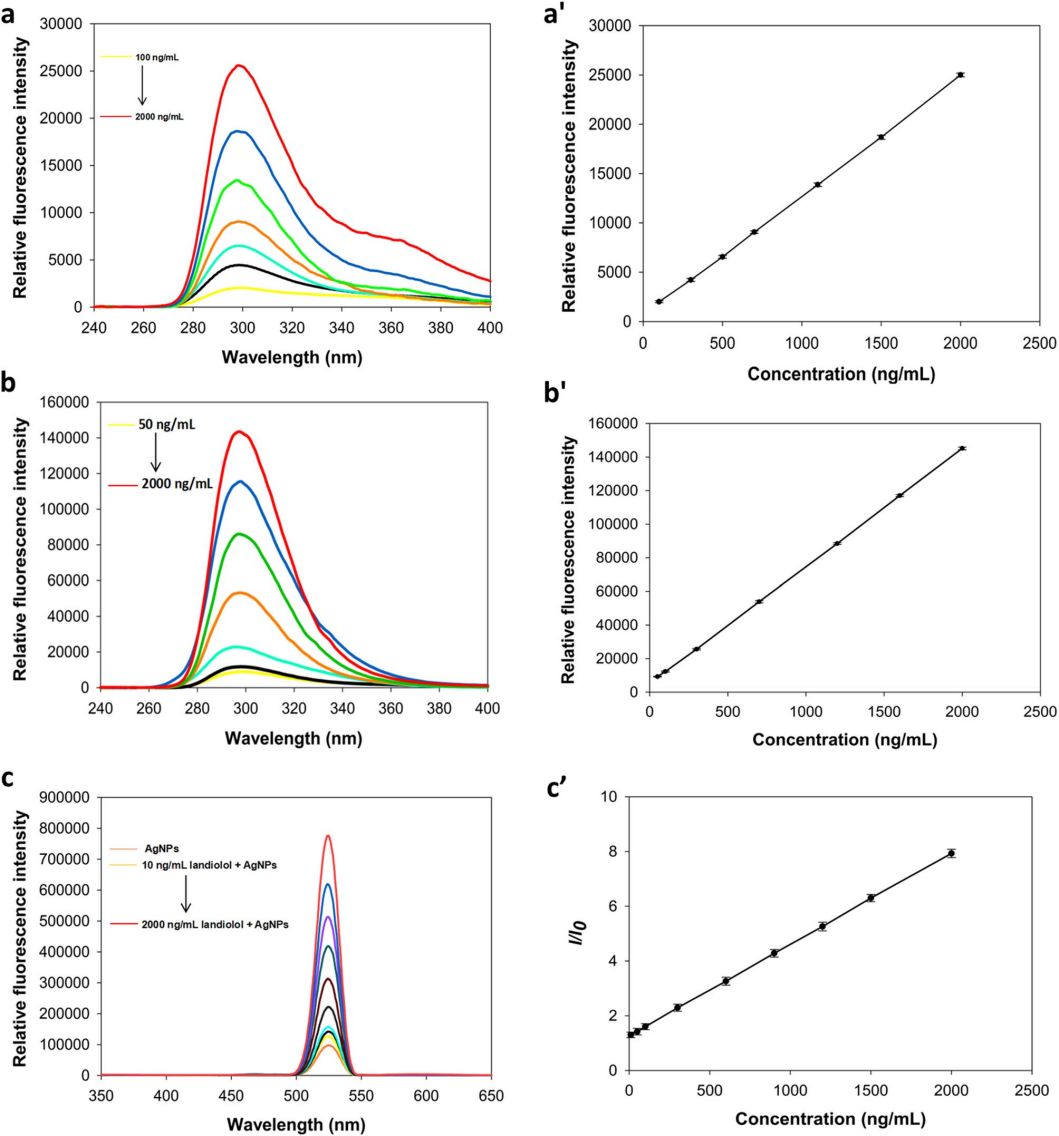
Emission spectra and corresponding calibration plots for (a, a’) method A, (b, b’) method B, and (c, c’) method C.

The proposed methods showed good linearity over the concentration ranges of 100–2000 ng/mL (*r* = 0.9998), 50–2000 ng/mL (*r* = 0.9999), 10–2000 ng/mL (*r* = 0.9999) of landiolol hydrochloride under the studied experimental conditions using method A, B and C; respectively (Fig. 6). To thoroughly characterize the analytical boundaries of AgNPs-based method, the broader dynamic concentration-response range of Method C was investigated up to 6000 ng/mL. Above 2500 ng/mL, the fluorescence enhancement gradually approached a plateau (3000–4500 ng/mL), indicating progressive saturation of the available binding sites on the AgNPs surface. At concentrations exceeding 4500 ng/mL, a slight decrease in fluorescence intensity was observed, which can be attributed to complete surface saturation together with optical effects arising from excess unbound analyte molecules in solution. Accordingly, the validated calibration range was restricted to 10–2000 ng/mL to ensure optimal linearity and accuracy. This also justified the implementation of a 5-fold dilution during plasma sample pretreatment (as detailed in Sect. 3.6), ensuring that clinical concentrations remained well within the validated linear range and sufficiently below the onset of saturation effects, thereby maintaining optimal analytical performance. The Limit of Detection (LOD) and Limit of Quantitation (LOQ) were determined using the signal-to-noise approach, calculated as 3.3 σ/S and 10 σ/S, respectively, where σ represents the standard deviation (SD) of the regression equation’s intercept and S denotes the calibration curve slope. The LOD values were determined to be 32.57, 16.31, 3.10 ng/mL, whereas the LOQ values were found to be 98.71, 49.41, 9.40 ng/mL indicating that method C is the most sensitive of the three proposed methods.

#### Accuracy and precision

Three different concentrations of landiolol hydrochloride covering the linearity range of each method were analyzed in triplicates. The selected concentrations for methods A, B, and C were (150, 800, and 1600 ng/mL); (150, 400, and 1500 ng/mL); (20, 700, and 1600 ng/mL), respectively. The accuracy was calculated as percentage recoveries. Recoveries of method A were in the range of 99.54% – 100.11%, recoveries of method B were in the range of 98.24% – 99.85% and recoveries of method C were in the range of 100.36% – 101.56% showing an excellent accuracy of the suggested methods as shown in Table 1. Methodological precision was evaluated by calculating the percent relative standard deviation (%RSD) for both intra-day repeatability (assessed within a single day) and inter-day intermediate precision (conducted over three consecutive days). The intra-day precision (%RSD) for Methods A, B, and C were 0.196, 0.684, and 0.213, respectively, while the inter-day precision (%RSD) values were 0.493, 0.823, and 0.379, as presented in Table 1.

#### Selectivity

Interference studies are crucial to assess selectivity because they verify that the proposed method can distinguish and quantify the analyte even in the presence of other interfering substances. The selectivity was evaluated by assessing the impact of common interferants that may be present in dosage form and biological fluids on the response of 100 ng/mL landiolol hydrochloride. The study examined excipients present in dosage form as mannitol, along with substances commonly found in plasma such as urea, creatinine, minerals, glucose, alanine, arginine, leucine. Aliquots of landiolol hydrochloride were mixed with interferants’ solution in a ratio (1: 10). The mixture was then analyzed as provided under (2.3.3) section. The fluorescence response exhibited a remarkable selectivity for landiolol, maintaining high signal integrity even in the presence of complex matrix interferents as shown in (**Fig. S4**).

The selectivity of the proposed methods was further assessed using blank human plasma samples subjected to the same protein precipitation and extraction procedure. The protein precipitation step efficiently removed the majority of plasma proteins and other macromolecular constituents that could potentially interfere with the fluorescence measurements. For Methods A and B, a low background fluorescence response arising from residual endogenous plasma constituents was observed at the analytical emission wavelengths. However, this response was substantially lower than that obtained for landiolol and was effectively corrected using appropriately processed blank plasma samples. For Method C, the longer emission wavelength (524.6 nm) significantly reduced the contribution of plasma autofluorescence and background scattering. The satisfactory recoveries and precision obtained for spiked plasma samples confirmed that residual matrix components did not adversely affect the determination of landiolol under the optimized conditions.

### Applications of proposed methods to dosage form and plasma

The suggested methods were successfully applied for quantifying landiolol hydrochloride in its dosage form. The Standard addition technique was performed by spiking the pre-analyzed dosage form (200 ng/mL) with landiolol hydrochloride at three distinct levels (50–150%). The proposed methods showed excellent recoveries, as presented in Tables 2 and 3.

**Table 2:** Determination of landiolol hydrochloride in its dosage form by the proposed methods.

Claimed (ng/mL)	Method A		Method B			Method C		
	Found^*^ (ng/mL)	%Recovery	Found^*^ (ng/mL)		%Recovery	Found ^*^ (ng/mL)		%Recovery
200	196.85	98.43	202.08		101.04	200.79		100.40
400	400.26	100.07	403.45		100.86	403.82		100.96
700	699.61	99.94	692.91		98.99	695.74		99.39
**Mean ± SD**	**99.48 ± 0.912**		**100.30 ± 1.135**			**100.25 ± 0.796**		

^*^ Each concentration was analyzed in triplicate.

**Table 3:** Determination of landiolol hydrochloride in dosage form by standard addition method.

Claimed (ng/mL)		Method A		Method B		Method C	
	Added (ng/mL)	Found^*^ (ng/mL)	%Recovery	Found^*^ (ng/mL)	%Recovery	Found^*^ (ng/mL)	%Recovery
200	100	101.29	101.29	99.40	99.40	100.79	100.79
200	200	200.64	100.32	201.12	100.56	199.27	99.64
200	300	299.89	99.96	299.02	99.67	300.79	100.26
**Mean ± SD**		**100.52 ± 0.688**		**99.88 ± 0.607**		**100.23 ± 0.576**	

^*^Each concentration was analyzed in triplicate.

To suppress potential esterase-mediated hydrolysis of landiolol ex-vivo, cold acetonitrile was immediately added upon plasma spiking to induce rapid both protein denaturation and precipitation as well as enzymatic quenching^5,36–38^, with all subsequent handling performed under cooled conditions immediately prior to measurement. Fluorescence measurements were also performed directly after sample preparation.

Based on reported clinical data, peak plasma concentrations (C_max_)^39^ of landiolol range from 520 to 1770 ng/mL in patients with atrial fibrillation/flutter and from 200 to 1000 ng/mL in healthy volunteers. The proposed plasma sample preparation procedure introduces an overall 5-fold dilution prior to analysis, resulting in corresponding analytical concentration ranges of approximately 104–354 ng/mL for patients and 40–200 ng/mL for healthy subjects. Accordingly, method A and method B are suited for monitoring steady-state and peak concentrations within the target patient population. Method C provides the widest analytical range and highest sensitivity, allowing reliable quantification across the full clinically relevant concentration window for both healthy subjects and patients. Furthermore, given the ultra-short elimination half-life of landiolol, plasma concentrations decline rapidly once intravenous infusion is discontinued. The enhanced sensitivity of Method C (LOD = 3.10 ng/mL and LOQ = 9.40 ng/mL) provides a distinct clinical advantage by enabling reliable detection of low residual concentrations during the post-infusion elimination phase. The recoveries for landiolol hydrochloride in plasma obtained by the three methods are presented in Table 4.

**Table 4:** Determination of landiolol hydrochloride in spiked human plasma by the proposed methods.

Spiked plasma concentration (ng/mL)	Method A		Method B		Method C	
	Found^*^ (ng/mL)	%Recovery	Found^*^ (ng/mL)	%Recovery	Found^*^ (ng/mL)	%Recovery
1770	1768.72	99.93	1766.42	99.80	1767.58	99.86
1000	987.95	98.80	987.5	98.75	1007.45	100.75
700	699.60	99.94	679.85	97.12	697.35	99.62
500	493.25	98.65	498.05	99.61	493.85	98.77
200	˂LOQ	---	˂LOQ	---	199.39	99.70
**Mean ± SD**	**99.33 ± 0.701**		**98.82 ± 1.222**		**99.74 ± 0.706**	

^*^Each concentration was analyzed in triplicate. Found concentrations reflect original plasma levels (corrected for a 5-fold extraction dilution).

### Greenness and blueness assessments

#### AGREE tool

The environmental performance of the proposed methods were quantitatively compared using the Analytical GREEnness (AGREE) metric software^40^, which is based on the twelve principles of green analytical chemistry. These principles include sample treatment, sample quantity, device positioning, number of sample-preparation steps, automation and miniaturization, derivatization, waste generation, analytical throughput, energy consumption, reagent sources, reagent toxicity, and operator safety. The software assigns a score between 0 and 1 for each principle, with the overall greenness score calculated as the average of these values. The results are presented in a circular pictogram, where each segment represents a criterion and is colored according to a traffic-light scale from red (least sustainable) to dark green (most sustainable). As shown in **Fig. S5**, methods A and C achieved a higher total score than method B, indicating that methods A and C are greener than method B. This difference is mainly attributed to the use of acetonitrile as the final diluting solvent in method B, which increases the overall environmental burden compared to the predominantly aqueous conditions used in methods A and C. In addition, method C exhibited a slightly lower AGREE performance compared to method A. This difference can be attributed to the inclusion of Ag NPs, which are automatically flagged by the AGREE metric under criteria related to reagent toxicity and environmental impact (Criteria 11 and 12), irrespective of the very small volume employed (5 µL). Despite this inherent limitation, the negligible quantity used, along with the green synthesis approach based on Aloe vera, ensures that the overall environmental impact remains minimal.

#### BAGI tool

The Blue Applicability Grade Index (BAGI), known by “blueness assessment” is a useful and quantitative tool for evaluating analytical methods based on their practicality. The index is built upon ten distinct criteria, covering every stage of the process, from the specific analysis type, complexity of instrumentation, number of analytes, required sample amounts, and preparation/preconcentration demands, to the throughput rate, availability of reagents and level of automation. The software yields two outputs. First a middle score ranging from 25 to 100, where a score of 25 indicates poor performance and 100 reflects excellent applicability. Second, a visual assessment via an asteroid pictogram, where increasing shades of blue points to greater applicability, and a white color denotes unsatisfactory results^41^. Methodological applicability was verified through the BAGI tool, where methods A and B demonstrated higher scores, likely due to their direct measurement, whereas method C requires additional synthesis step. It should be noted that method C is the most sensitive of all the three methods, but the BAGI tool does not consider the LOD in its assessment (**Fig. S6**).

### Comparison with reported methods

To the best of our knowledge, no optical analytical method has previously been reported for the determination of landiolol hydrochloride, making the present work the first fluorimetric approach developed for its analysis.

The proposed fluorimetric methods were compared with previously reported chromatographic methods for the determination of landiolol hydrochloride. As summarized in Table S1, the comparison includes key analytical parameters, including linearity range, sensitivity (LOD/LLOQ), sample preparation requirements, analysis time, and greenness assessment based on the AGREE metric. The results demonstrate that the proposed methods offer significant advantages in terms of simplicity, rapidity, and environmental sustainability, while providing satisfactory analytical sensitivity for the determination of landiolol hydrochloride in pharmaceutical formulations and human plasma.

## Conclusion

A stepwise fluorimetric approach was successfully developed for the sensitive and rapid determination of landiolol hydrochloride. To the best of our knowledge, this work represents the first fluorimetric approach reported for the analysis of landiolol hydrochloride in dosage forms and human plasma. The three methods demonstrated a progressive improvement in analytical performance: the native fluorescence in water offered a simple, cost-effective, and eco-friendly approach with LOD of 32.57 ng/mL; measurement in acetonitrile enhanced sensitivity reaching LOD of 16.31ng/mL; and the AgNPs-based method achieved the highest sensitivity (LOD of 3.10 ng/mL) while maintaining a green and sustainable profile through the use of *Aloe vera*-mediated nanoparticles. Greenness and blueness assessments using the AGREE and BAGI tools further confirmed the environmental and practical sustainability of the proposed approaches. Collectively, these methods provide a versatile toolkit for landiolol analysis, balancing sensitivity, simplicity, and environmental sustainability.

## Supplementary Information

Below is the link to the electronic supplementary material.

Supplementary Material 1

## Data Availability

The datasets generated and/or analyzed during the current study are available on reasonable request.
