# Supplementary material for: Comparative fluorimetric strategies for determination of landiolol hydrochloride using native fluorescence and green synthesized silver nanoparticles
Source: Sci Rep. 2026 Jul 28;16:23495. doi: 10.1038/s41598-026-63573-1 (PMC13415514; doi:10.1038/s41598-026-63573-1)
Supplement: Supplementary file 1 — Supplementary Material 1 [file 41598_2026_63573_MOESM1_ESM.docx]

**Supplementary material**

**Comparative Fluorimetric Strategies for Determination of Landiolol Hydrochloride Using Native Fluorescence and Green Synthesized Silver Nanoparticles**

Marwa Khaled*^a^*, Hend Z. Yamani*^a*^*, Nermine V. Fares*^a^*, Amira M. El-Kosasy*^a^*

*^a^ Pharmaceutical Analytical Chemistry Department, Faculty of Pharmacy, Ain Shams University, Cairo 11566, Egypt*

*Corresponding Author: Hend Z. Yamani

Email: [hend.z.yamani@pharma.asu.edu.eg](mailto:hend.z.yamani@pharma.asu.edu.eg)


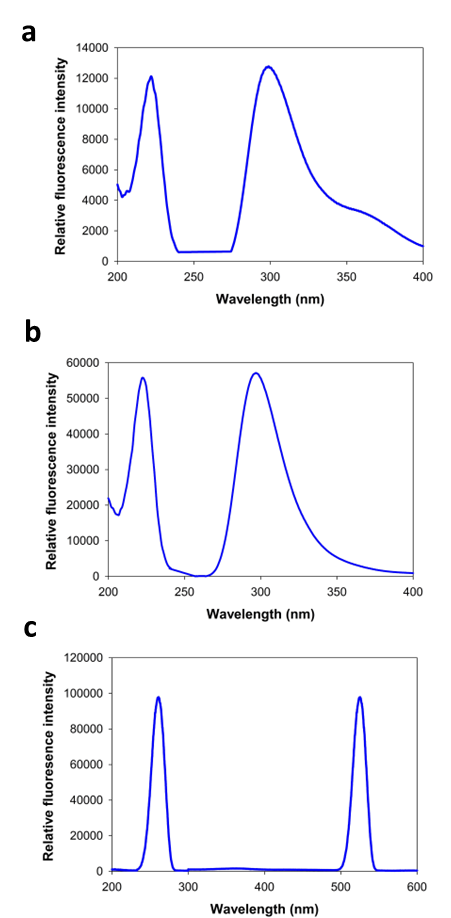


**Fig. S1.** Excitation and emission spectra of (a) landiolol hydrochloride in water,

(b) landiolol hydrochloride in acetonitrile, (c) AgNPs


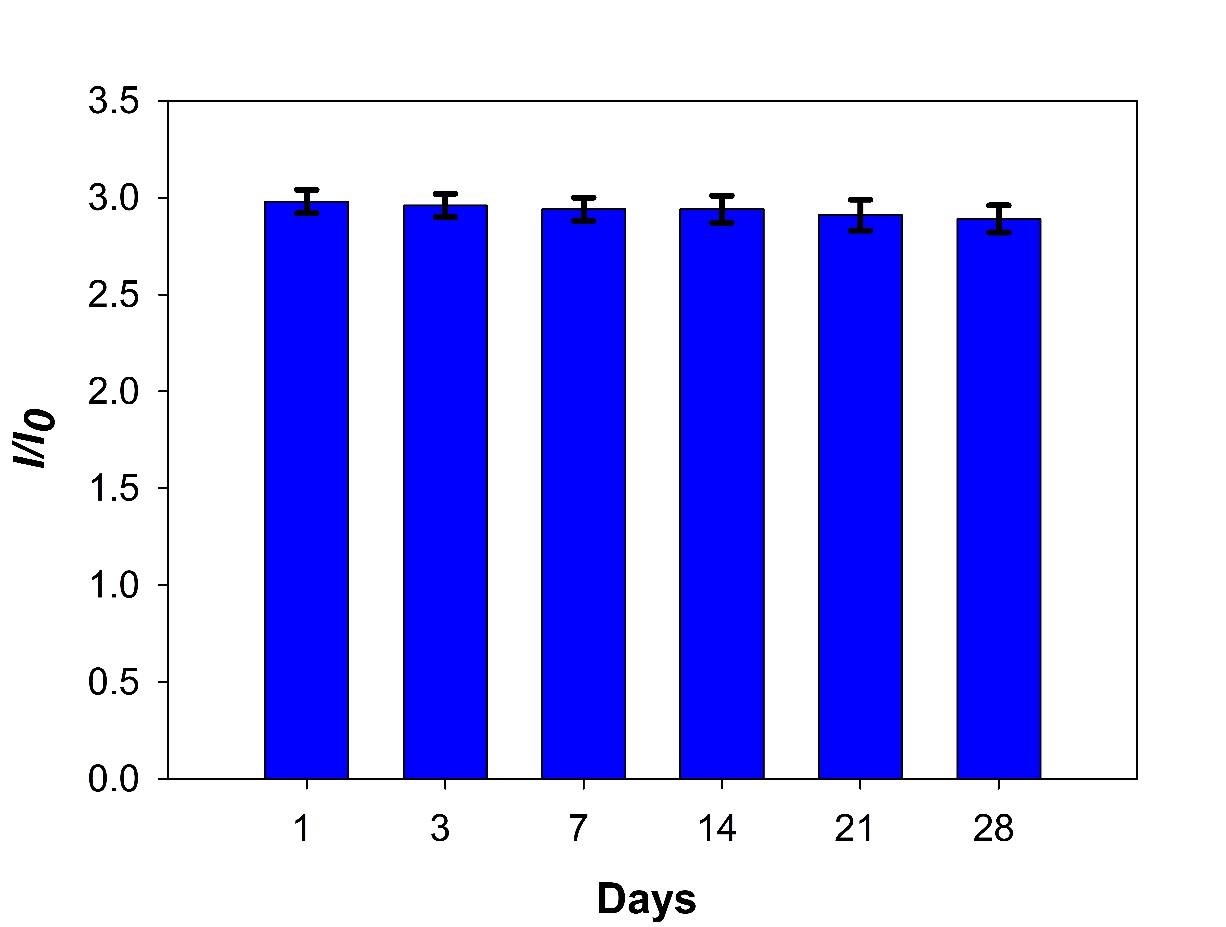


**Figure S2.** Stability of green synthesized AgNPs stored at 4 °C in the dark, expressed as relative fluorescence enhancement (I/I₀) of the landiolol–AgNP system using 500 ng/mL landiolol hydrochloride.


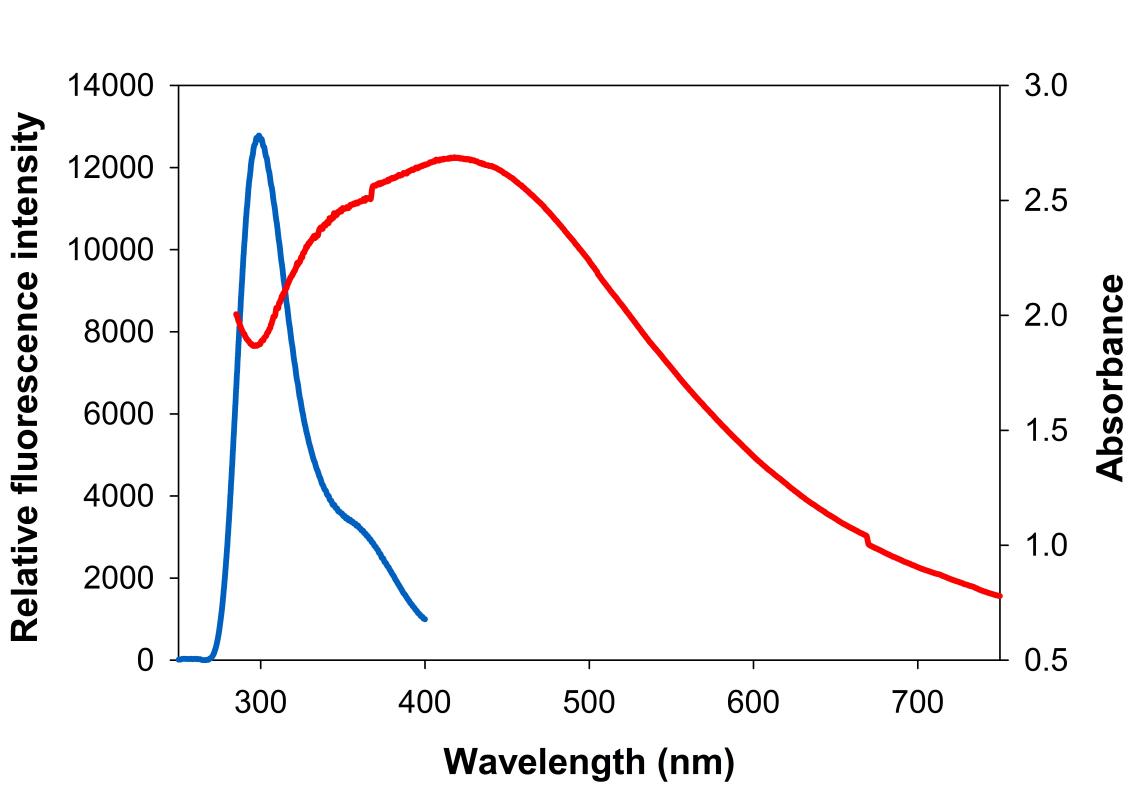


**Fig. S3.** Spectral overlap between the fluorescence emission spectrum of landiolol (blue) and the UV-Vis absorption spectrum of AgNPs (red).


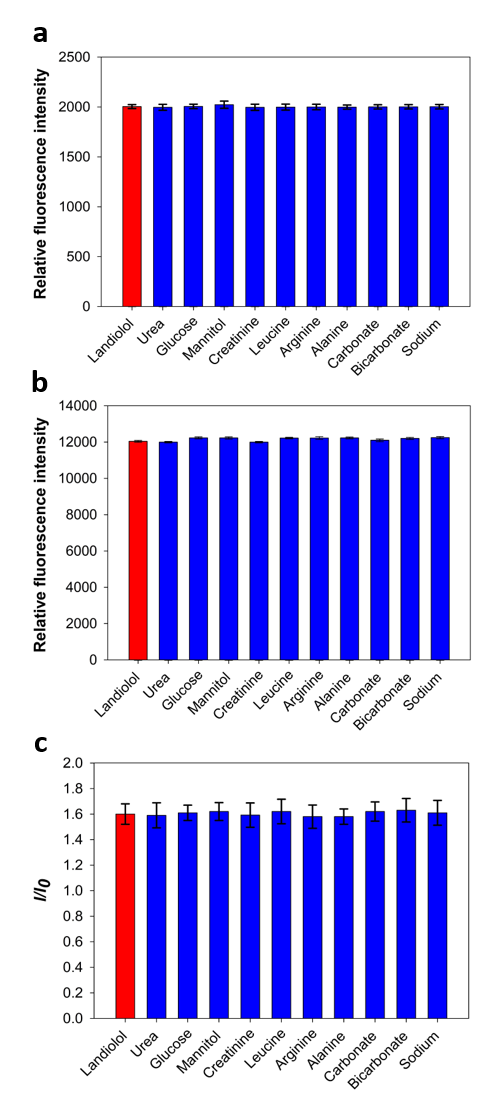


**Fig. S4**. Effect of different interferants on the relative fluorescence intensity of 100 ng/mL landiolol hydrochloride using (a) method A, (b) method B, and (c) method C.


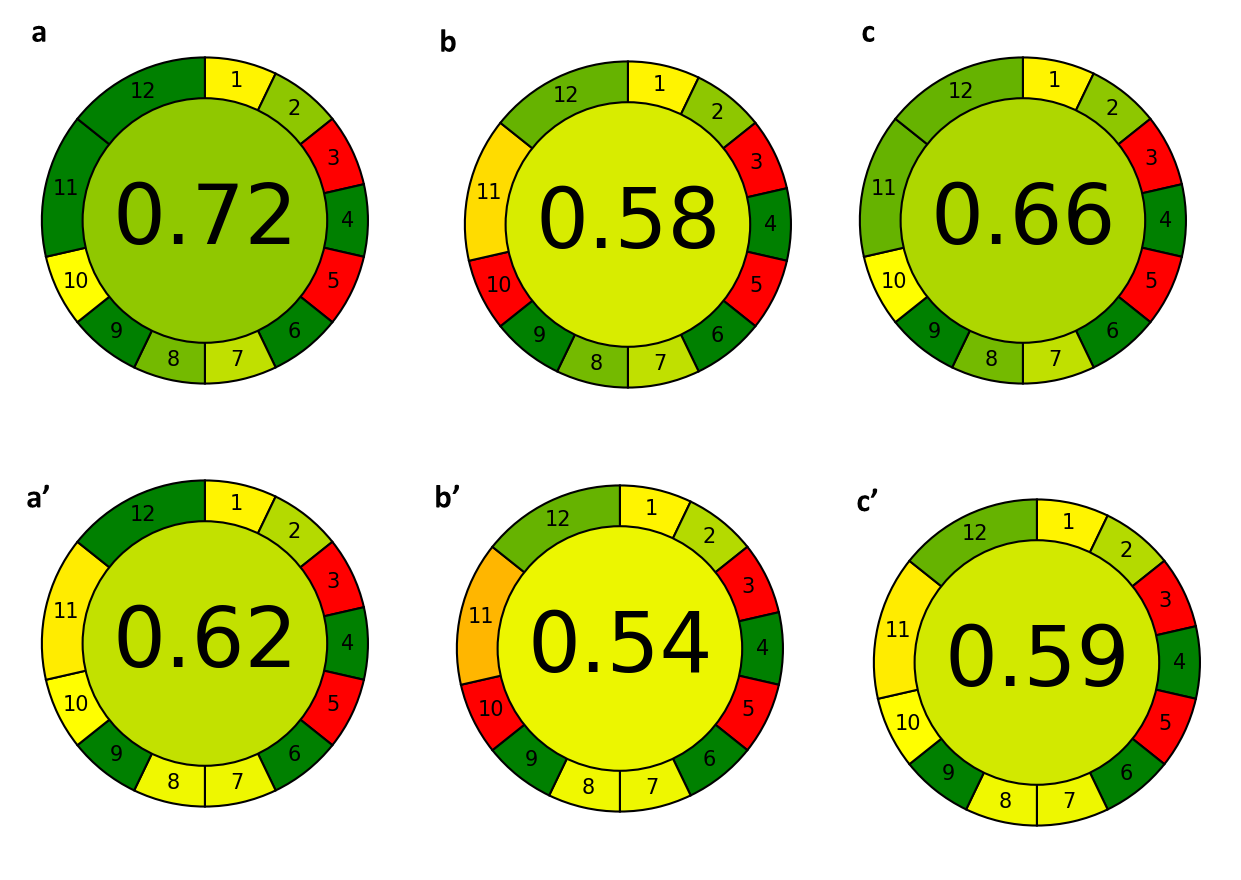


**Fig. S5.** AGREE greenness assessment of methods A, B, and C applied to dosage forms and plasma, represented by (a–a′), (b–b′), and (c–c′), respectively.


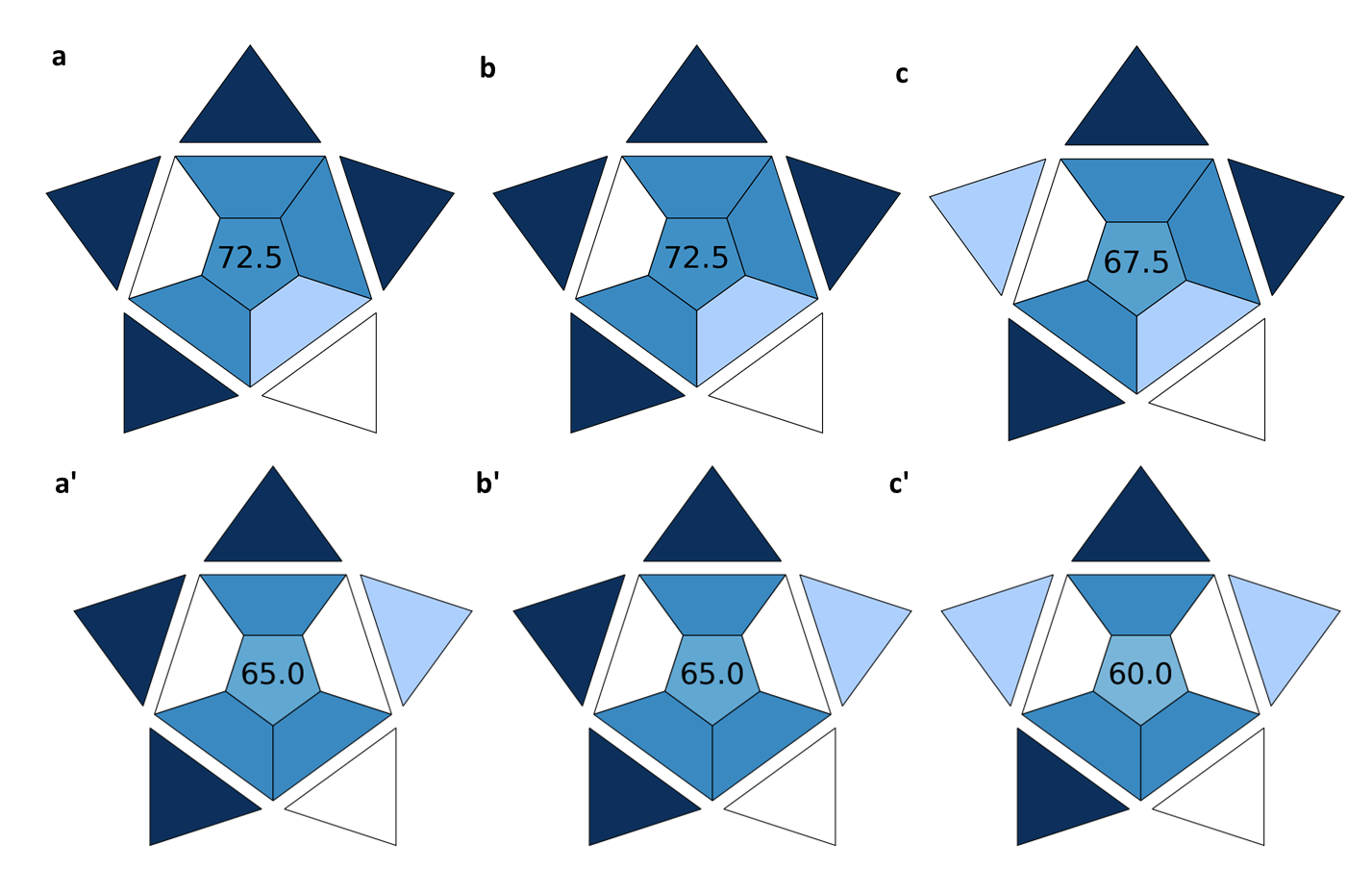


**Fig. S6.** Blueness assessment using BAGI tool for methods A, B, and C applied to dosage forms and plasma, represented by (a–a′), (b–b′), and (c–c′), respectively.

**Table S1.** Comparison between the proposed methods and reported methods for landiolol determination

| **Methods** | **Linear range (ng/mL)** | **LOD** | **LOQ/LLOQ** | | **Biological sample preparation** | **Analysis time (min)/run** | **AGREE score** | **Applications** | **Ref.** |
| --- | --- | --- | --- | --- | --- | --- | --- | --- | --- |
|  |  | **(ng/mL)** | | |  |  |  |  |  |
| HPLC-UV | 50-10,000 | --- | | 50 | PPT + SPE + LLE with intermediate drying steps | 35 | 0.30 | Human blood | [5] |
| HPLC-fluorescence | 10-5000 | --- | | 10 | PPT + SPE + evaporation/ reconstitution | 18 | 0.30 | Human blood | [6] |
| HPLC-fluorescence | 100-10,000 | 50 | | 100 | LLE + evaporation/ reconstitution | 9 | 0.44 | Human plasma | [7] |
| HPLC-MS/MS | 0.5-500 | --- | | 0.5 | LLE + evaporation/ reconstitution | 3.5 | 0.43 | Human plasma | [8] |
| Method A | 100-2000 | 32.57 | | 98.71 | Dosage form: Direct dilution after reconstitution  Plasma: PPT + evaporation and reconstitution | ˂1 | Dosage form: 0.72  Plasma: 0.62 | Dosage form and human plasma | This work |
| Method B | 50-2000 | 16.31 | | 49.41 | Dosage form: Direct dilution after reconstitution  Plasma: PPT + evaporation and reconstitution | ˂1 | Dosage form: 0.58  Plasma: 0.54 | Dosage form and human plasma | This work |
| Method C | 10-2000 | 3.10 | | 9.40 | Dosage form: Direct dilution after reconstitution  Plasma: PPT + evaporation and reconstitution | ˂1 | Dosage form: 0.66  Plasma: 0.59 | Dosage form and human plasma | This work |

PPT: Protein Precipitation; SPE: Solid -Phase Extraction; LLE: Liquid-Liquid Extraction
